# A Case of Profound Hypertriglyceridemia Causing Pseudohypobicarbonatemia

**DOI:** 10.7759/cureus.37489

**Published:** 2023-04-12

**Authors:** Deepshikha Garg, Tulika Chatterjee

**Affiliations:** 1 Department of Internal Medicine, University of Illinois, Peoria, USA

**Keywords:** acid-base disorders, spurious lab, factitious hypobicarbonatemia, severe hypertriglyceridemia, high anion gap metabolic acidosis

## Abstract

The light-scattering effect of hypertriglyceridemia may interfere with the photometric analysis of the electrolytes, leading to errors in laboratory values. We present a case of erroneously low bicarbonate levels due to the presence of severe hypertriglyceridemia. A 49-year-old male was admitted for knee cellulitis. A comprehensive metabolic panel showed very low bicarbonate of <5 mmol/L, and an elevated anion gap of 26 mmol/L. The lactic acid, salicylic acid, ethanol, and methanol levels were normal. The lipid panel showed a remarkably high triglyceride level of 4846 mg/dL. An arterial blood gas (ABG) showed a normal pH of 7.39 and a bicarbonate level of 28 mmol/L, which was inconsistent with the metabolic acidosis seen in the blood test. The discrepancy between acidosis seen in the metabolic panel and ABG was explained by a lab error in the measured bicarbonate levels, which occurs in the presence of elevated triglyceride levels. Most laboratories use either an enzymatic/ photometric or an indirect ion-selective electrode method to measure bicarbonate. Hyperlipidemia interferes with photometric analysis due to its light-scattering effect. An ABG analyzer uses a direct ion-selective electrode method that is free of the errors of a photometric analyzer. Knowing about conditions like hypertriglyceridemia, which can interfere with the measurement of electrolytes, is important in everyday clinical medicine, as it can prevent unnecessary investigation and intervention.

## Introduction

High anion gap metabolic acidosis (HAGMA) is often associated with life-threatening conditions like sepsis, severe lactic acidosis, and alcoholic/diabetic ketoacidosis and should be identified and acted upon promptly [1]. However, there are certain conditions that can lead to falsely low bicarbonate levels on the metabolic panel. The light scattering effect of hypertriglyceridemia or paraproteinemia may interfere with the laboratory photometric analysis of the electrolytes leading to profound errors in laboratory values and thus causing substantial confusion for the clinician [2]. We present a case of erroneously low bicarbonate levels due to severe hypertriglyceridemia. In case of very low bicarbonate levels without an obvious cause, arterial blood gas (ABG) should be considered. ABG uses blood pH and partial pressure of carbon dioxide to calculate the bicarbonate values using the Henderson-Hasselbalch equation [1]. If there is a discrepancy between the reads on ABG and the metabolic panel, the presence of hypertriglyceridemia should be considered a confounding factor.

## Case presentation

A 49-year-old male with a history of hypertension, diabetes mellitus type 2, diabetic neuropathy, hypothyroidism, hyperlipidemia, and coronary artery disease presented to the emergency department with complaints of left suprapatellar pain and swelling. He denied any recent fevers, myalgia, shortness of breath, chest pain, or abdominal pain. The only medication he was taking was metformin, and he was not taking any of his other prescribed medications, including aspirin, gabapentin, glimepiride, insulin glargine, levothyroxine, atorvastatin, and gemfibrozil, due to financial constraints. He did have a smoking history of 25 pack years. His family history was positive for heart failure and hyperlipidemia in his father. On admission, his BMI was noted to be 38.35 kg/m^2^ and his blood pressure was 166/100 mmHg, the remaining vitals were within normal range. The left lower extremity exam was consistent with cellulitis over the supra-patellar region.

Initial laboratory workup showed low bicarbonate of <5 mmol/L, and elevated anion-gap of over 26 mmol/L. Other initial blood work is tabulated in Table 1. Additional workup for HAGMA showed a mildly elevated creatine kinase (CK) at 1482 U/L while lactic acid, salicylic acid, ethanol, and methanol levels were within normal limits (Table 1).

**Table 1:** Laboratory workup on admission BUN: blood urea nitrogen; GFR: glomerular filtration rate; HbA1c: glycated hemoglobin; VLDL: very-low-density lipoprotein

Laboratory test	Laboratory values	Reference range and Units
Hemoglobin	14.9	13 - 16.6 g/dL
White blood count	10.37	4 - 12 10(3)/mcL
Sodium	128	136 - 145 mmol/L
Potassium	5.0	3.5 - 5.1 mmol/L
Chloride	97	98 - 107 mmol/L
CO2, venous	<5	22 - 30 mmol/L
Anion gap	>26	<18 mmol/L
Glucose	244	70 - 99 mg/dL
BUN	23	8 - 26 mg/dL
Creatinine	1.54	0.70 - 1.30 mg/dL
BUN/Creatinine ratio	15	12 - 20 ratio
GFR, Est. Non-African	48	>=60 mL/min/1.73m2
Creatine kinase	1482	30 - 200 U/L
Thyroid-stimulating hormone	316.318	0.3 - 5 mIU/L
Free T3	1.9	1.7 - 3.7 pg/ml
Free T4	0.4	0.7 - 1.9 ng/dL
HbA1c	9.8	4 - 6 %
Lactic acid	1.7	0.7 - 2.0 mmol/L
Salicylate level	<5	15 - 30 mg/dL
Ethanol level	<10	<10 mg/dL
Methanol level	<10	<10 mg/dL
Urine ketones	Negative	Negative
ABG		
pH Arterial	7.39	7.35 - 7.45
pCo2 Arterial	44	35 - 45 mmHg
pO2 Arterial	80	85 - 105 mmHg
Total Bicarb (tCo2)	28	23 - 27 mmol/L
Lipid panel		
Total cholesterol	798	<200 mg/dL
LDL	Unable to calculate LDL when triglycerides>400	<130 mg/dL
HDL	28	>40 mg/dL
Non-HDL cholesterol	770	<130 mg/dL
Triglycerides	4846	<150 mg/dL
VLDL	Unable to calculate LDL when triglycerides >400	10 - 50 mg/dL

Intravenous normal saline with bicarbonate was initiated on admission due to low bicarb, though the patient remained asymptomatic. On admission to the floor, an arterial blood gas (ABG) showed a normal pH of 7.39 and a bicarbonate level of 28 mmol/L, which was inconsistent with the severe metabolic acidosis seen in the complete metabolic panel (CMP) (Table 1). Serial CMPs kept showing HAGMA (Table 2). The bicarbonate drip was subsequently stopped given the absence of clinical acidosis symptoms and normal ABG.

**Table 2:** Serially drawn CMPs showing HAGMA CMP: complete metabolic panel; HAGMA: high anion gap metabolic acidosis

Component	Ref Range and Units	6/30/2020	6/30/2020	6/30/2020	7/1/2020	7/1/2020	7/2/2020
		01:14 am	6:15 am	2:55 pm	9:36 am	7:06 pm	8:09 am
Sodium	136 –145 mmol/L	128	131	128	127	127	128
Potassium	3.5 -5.1 mmol/L	5.0	4.3	4.2	4.1	4.3	4.3
Chloride	98 –107 mmol/L	97	99	91	95	94	95
CO2, venous	22 –30 mmol/L	<5	14	16	<5	11	12
Anion gap	<18 mmol/L	>26	18	21	>27	22	21

Meanwhile, the lipid panel showed a high total cholesterol of 798 mg/dL and a remarkably high triglyceride level of 4846 mg/dL (Table 1). The discrepancy between bicarbonate levels seen in CMP and ABG was explained by an erroneous measurement of bicarbonate levels, which happens in the presence of severely elevated triglyceride levels. Severe hypertriglyceridemia can affect the measurement of bicarbonate when using the indirect ion-selective measuring system, which can lead to reporting of pseudohypobicarbonatemia as was seen in this patient.

Since he remained asymptomatic from hypertriglyceridemia, and there were no signs or symptoms of acute coronary syndrome or pancreatitis, insulin drip or plasmapheresis was not initiated. The patient’s high triglyceride levels were likely from familial hypertriglyceridemia, severe hypothyroidism, and statin non-compliance. The mildly elevated CK levels were also thought to be from severe hypothyroidism. His levothyroxine was resumed while statin and fibrates were held on discharge for CK levels to normalize before resuming them outpatient, due to the increased risk of rhabdomyolysis with statins in the setting of elevated CK. Case management helped in procuring medications for the patient on discharge. He was discharged home after four days of hospitalization in a stable condition with close follow-up with his primary care physician. An outpatient metabolic panel, CPK level, lipid panel, and TSH were ordered but unfortunately, the patient was lost to follow-up. He was readmitted six months later with abdominal pain and triglyceride (TG) levels above 5000 mg/dl and resultant pseudohypobicarbonatemia. He required an insulin drip this time. High-dose statin and fenofibrate were initiated inpatient this time as his CPK was normal. After one year of his initial hospitalization, his TG level was 324 mg/dl.

## Discussion

Acid-base disorders are frequently encountered in clinical practice. Determining the nature of the acid-base disorder, in association with the clinical presentation can narrow down the differentials and the plausible underlying cause and help guide appropriate therapy [3]. Precise measurement of serum bicarbonate concentration is required for the assessment of acid-base disorders [4]. Two different estimates are commonly used for measuring bicarbonate levels. One is a direct measurement from the metabolic panel, and the other one reports a derived bicarbonate value from ABG [5].

The first method of bicarbonate measurement via the metabolic panel is based on the amount of total carbon dioxide (T-CO2) in the blood. T-CO2 consists of 95% of bicarbonate while dissolved CO2 and carbonic acid constitute the remaining ~5%. Thus, measuring T-CO2 is a reasonably accurate estimate of the serum bicarbonate level [5,6]. For measuring T-CO2, automated chemistry analyzers use either an electrode-based/indirect ion-selective electrode (ISE) or the enzymatic/ spectro-photometric method [2]. The Beckman Coulter Unicel DxC analyzer (Brea, California) uses the indirect ISE method [7]. The Abbott Architect analyzer (Abbott Laboratories, Abbott Park, Illinois) uses the spectro-photometric method. When white light passes through a solution containing the analyte, certain wavelengths get absorbed (based on the specific properties of the analyte). For example, hemoglobin absorbs light in the green range of the spectrum (wavelength 500-600 nm), making the solution appear red. The decrease in green light is proportional to the amount of hemoglobin present. Some substances have no visible color and absorb light in the ultraviolet region. Based on this concept, a decrease in light absorbance between 404 and 410 nm, as measured via a photometric cell, is proportional to the T-CO2 content of the blood sample [8,9].

The second method for bicarbonate estimation using ABG analysis is based on the Henderson-Hasselbalch (HH) equation, where pH is the acidity in the blood (HCO−3) is the concentration of bicarbonate in the blood in mmol/Liter and PCO2 is the partial pressure of carbon dioxide (CO2) in the blood in mmHg. From the patient’s ABG sample, pH and pCO2 are measured using a direct ISE analyzer, and bicarbonate is then calculated using the HH equation above [1,7,10].

Typically, the serum bicarbonate levels measured by using the T-CO2 method and calculated by the gas analyzer method are found to be comparable (within 10% of each other) and interchangeable for clinical purposes [3]. However, the disparity between the serum bicarbonate levels from the two methods can be due to numerous reasons like dilution of plasma by heparin volume, venous CO2 accumulation due to tourniquet-induced stasis, and loss of CO2 from serum due to underfilling of vacuum collection, to mention a few [6]. Additionally, the enzymatic assay can interfere with wavelength measurement due to the presence of substances affecting serum turbidities such as bilirubin, hemoglobin, paraproteins, and lipids [11]. Hyperlipidemia also has a space-occupying effect, decreasing the aqueous phase of the sample, which can cause artifacts using both the indirect ISE and the enzymatic methods [7]. The Beckman Coulter assay claims minimal interference at < 1000 mg/dL of Intralipid (a soy-based lipid emulsion) concentration, but there is no warranty of bicarbonate accuracy above this level; and the Abbott Architect analyzer recognizes interference with T-CO2 measures in a dose-response manner, with an Intralipid concentration of >2000 mg/dL [7,9]. It is worth noting that patients with similar levels of hypertriglyceridemia can have huge variations in the size of lipid particles, resulting in varying degrees of photometric interference [9].

Goldwasser et al. reported a patient with extremely low T-CO2 and measured serum bicarbonate and HAGMA, but normal plasma bicarbonate was calculated using the ABG, with a normal to slightly alkaline arterial pH, persisting throughout a five-month period. The discrepancy was attributed to the presence of para-proteins resulting in increased serum turbidity and a consequent change in light absorbance, resulting in falsely low measured T-CO2 levels [6].

Rifkin et al. described the first case of pseudohypobicarbonatemia caused by profound hyperlipidemia. Using the Abbott Architect analyzer, the patient’s lab work showed a serum bicarbonate level <5 mEq/L while it was normal on ABG. The triglyceride level was >7000 mg/dL. It was postulated that the light-scattering effect of hyperlipidemia interfered with the enzymatic method, resulting in factitious low bicarbonate measures and HAGMA. This incongruity in measurements of bicarbonate levels resolved after treatment with lipid-clearing agents [7]. Since then, many cases showing discord between ABG-calculated and auto-analyzer-measured values of serum bicarbonate, associated with hyperlipidemia, have been reported [1,9,11-13].

Varghese et al. analyzed laboratory data of patients with TG greater than 1000 mg/dl on admission, serum bicarbonate level of less than 12 mEq/L, and had an ABG done with six hours of venous sampling. The results showed 60% of the instances of low bicarbonate levels in severe hypertriglyceridemia were due to spurious laboratory analyses due to lipemic interference. The level of TG correlated with the magnitude of the bicarbonate measurement error (r = 0.59; P < 0.001) [12].

Lipid interference can be avoided using ultracentrifugation, high-speed centrifugation, or a lipid-clearing agent, such as LipoClear, which could not be performed in our laboratory. The preferred final triglyceride concentration in the cleared sample should be <15 mmol/L [7]. Varghese et al. noted in their study that ultracentrifugation, and not simple centrifugation, may prevent lipid interference [12].

Plasmapheresis or therapeutic plasma exchange (TPE) is another efficient way to reduce serum triglyceride levels, however, evidence to support its use in improving clinical outcomes is limited [14]. According to the American Society of Apheresis guidelines, TPE is listed as a category III treatment recommendation for hypertriglyceridemia-induced acute pancreatitis. Its use (versus medical management alone with IVF and/or insulin) is physician and facility-dependent [11,15]. Carag et al. were among the first to use TPE to aggressively manage five patients who had severe hypertriglyceridemia and presented with or were at risk for severe pancreatitis and who also had notable pseudohypobicarbonatemia. The TPE-induced rapid reduction in triglyceride levels led to the immediate correction of the discrepant lab findings [11].

In our case, the patient was found to have very low bicarbonate and HAGMA on multiple metabolic panels but blood pH and calculated bicarbonate were normal on the ABG. Our lab uses a GEM analyzer, which is an enzyme-based/photometric analyzer. It was then ascertained that the elevated triglyceride levels lead to falsely low or pseudohypobicarbonatemia. The delay in recognizing hypertriglyceridemia-induced pseudohypobicarbonatemia did result in a futile workup for HAGMA, as well as initiation of bicarbonate drip. Current guidelines recommend intravenous bicarbonate therapy for treating acute metabolic acidosis if the pH is < 7.1 or 7.1-7.2 in the presence of severe acute kidney injury [16]. Our patient was not symptomatic of hypertriglyceridemia and did not need rapid lipid lowering using ultracentrifugation, lipid clearing agents, or TPE.

Accurate clinical evaluation of patients can prevent confusion created by spurious laboratory values. Diabetics often have concomitant hypertriglyceridemia, and are predisposed to various forms of acidosis (e.g., diabetic acidosis), making it more challenging to arrive at the correct metabolic abnormality [17]. In addition to physician knowledge of this artifact caused by hypertriglyceridemia, several measures proposed to avoid this oversight include clinician awareness about the analyzer being used at their hospital lab [9], promoting more frequent and timely use of ABGs to confirm a low bicarbonate level, automated reporting of high lipemic indices, and use of lipid-lowering treatment/s prior to measurement of T-CO2 [7,17].

## Conclusions

This case demonstrates the clinical implication of a laboratory error that occurs with certain analyzers. Our case emphasizes that physician knowledge about factitious hypobicarbonatemia from hypertriglyceridemia can prevent misdiagnosis and unnecessary treatment with potential side effects. This entity should be considered when there is no discernible cause for low bicarbonate, and ambiguity is noted between bicarbonate levels measured on metabolic panels and calculated by ABGs.
